# Immunotherapy of hepatocellular carcinoma with infection of hepatitis B or C virus

**DOI:** 10.20517/2394-5079.2020.58

**Published:** 2020-10-12

**Authors:** Cecilia Monge Bonilla, Nicole A. McGrath, Jianyang Fu, Changqing Xie

**Affiliations:** Thoracic and Gastrointestinal Malignancies Branch, Center for Cancer Research, National Cancer Institute, National Institutes of Health, Bethesda, MD 20892, USA

**Keywords:** Hepatocellular carcinoma, hepatitis B virus, hepatitis C virus, immunotherapy

## Abstract

Hepatocellular carcinoma (HCC) has one of highest mortalities globally amongst cancers, but has limited therapeutic options once in the advanced stage. Hepatitis B or C virus infection are the most common drivers for HCC carcinogenesis, triggering chronic liver inflammation and adding to the complexity of the immune microecosystem of HCC. The emergence of immunotherapy has afforded a new avenue of therapeutic options for patients with advanced HCC with a history of hepatitis B or C virus infection. This article reviews the change of immunity elicited by hepatitis B or C virus infection, the immune feature of HCC, and the clinical evidence for immunotherapy in advanced HCC and discusses future directions in this field.

## INTRODUCTION

Liver cancers are the fourth leading cause of cancer-related mortality worldwide^[1,2]^, and there are over 800,000 new primary liver cancer cases around the world each year^[3]^. Hepatocellular carcinoma (HCC) accounts for 75%−85% of these cases and is one of the most aggressive liver cancers^[1]^. The incidence of HCC is increasing in many high-income countries^[2]^. The majority of HCC occurs in patients with underlying chronic liver diseases triggered by various risks dependent on geographic area, sex, age, and degree of liver damage^[4]^. Furthermore, males are twice as likely as females to develop HCC^[5]^.

HCC can be caused by both viral and non-viral factors. HCC develops secondary to chronic infection with the hepatitis B virus (HBV) or hepatitis C virus (HCV). High serum levels of HBV DNA and HCV RNA viral load are considered to be independent risk factors for developing HCC in patients infected by these diseases^[6,7]^. HBV vaccination has greatly reduced the incidence of HCC in certain geographic areas^[8]^. Moreover, improved screening and treatment of HCV infection has also reduced virus-related HCC cases in non-epidemic regions^[9]^.

Non-viral risk factors for the development of HCC include excessive alcohol consumption, environmental exposure to aflatoxin, metabolic disorders, non-alcoholic steatohepatitis, and genetic disorders^[10]^. It is unsurprising that non-viral risk factors are more common causes of HCC in countries such as the USA, UK, and other high income countries. Frequently, viral infection is complicated with non-viral risk factors leading to HCC development. Systematic treatment is the standard approach to control advanced HCC, given that most patients present with advanced stage disease, which limits curative approaches such as surgical resection, liver transplantation, and local liver-directed therapy. Recent molecular landscape analysis has led to the development of systematic targeted therapies for advanced HCC, including sorafenib^[11]^ and lenvatinib^[12]^ in the first line setting, and regorafenib^[13]^, cabozantinib^[14]^, and ramucirumab^[15]^ as second line options. The breakthrough of cancer immunology research has provided effective immunotherapy by blocking immunosuppressive mechanisms and enhancing host immune surveillance. This leads to the recognition of tumour and execution of a tumour-specific response capable of treating malignancy, including HCC^[16]^. HBV- or HCV-related HCC represents a special entity compared to non-viral HCC. This review discusses the immune response to HBV and HCV infection, the immunology of HCC, and summarizes the current status of immunotherapy in HCC in the context of HBV or HCV infection.

## HBV INFECTION AND IMMUNE TOLERANCE

**Figure 1.**
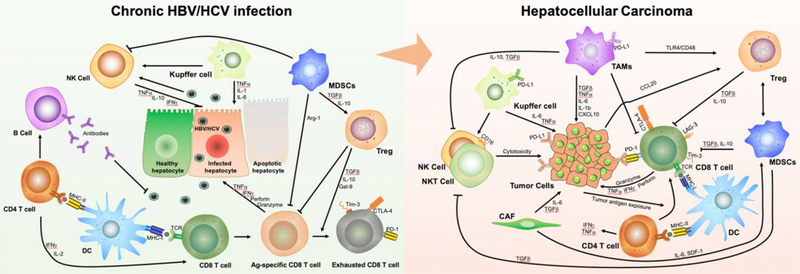
Schematic mechanism of immune evasion across the spectrum from inflammation by chronic hepatitis B (HBV) and C virus (HCV) infection to resultant hepatocellular carcinoma (HCC). The complexity of the mechanism involves multiple immune cells and various collections of cytokines. A: immune tolerance induced by HBV and HCV infection; B: immune evasion driven by the crosstalk between tumour cells and immune cells in HCC. DC: dendritic cells; MDSCs: myeloid-derived suppressor cells; TAMs: tumour-associated macrophages; NK: natural killer; NKT: natural killer T; Treg: regulatory T-cells; CAF: cancer-associated fibroblast; HBV: hepatitis B virus; HCV: hepatitis C virus; MHC: major histocompatibility complex; TCR: T-cell receptor; IL: interleukin; IFNg: interferon gamma; TNFa: tumour necrosis factor receptor alpha; TGF-β: transforming growth factor beta; CCL: C-C motif chemokine ligand; CXCL: C-X-C motif ligand 1; Gal-9: galactin-9; PD-1: programmed cell death protein; PD-L1: programmed cell death ligand 1; CTLA: cytotoxic T-lymphocyte-associated protein; IL: interleukin; Arg-1: arginase-1; Tim-3: T cell immunoglobulin and mucin domain 3; LAG-3: lymphocyte-activation gene 3; SDF-1: Stromal cell-derived factor 1

Studies have shown that HBV not only has a direct carcinogenic effect through the integration of viral DNA and the oncoprotein HBV-encoded X protein (HBx), but also has an indirect carcinogenic effect due to chronic immune suppression^[17]^. HBV has been considered as a stealth virus and acute infection does not lead to a strong activation of interferon (IFN) and pro-inflammatory responses^[18–22]^. Liver resident macrophage Kupffer cells are able to interact with hepatitis B surface antigen (HBsAg) and produce pro-inflammatory cytokines, but Toll-like receptor expression is down-regulated by HBeAg^[23,24]^. Indirect activation of natural killer (NK) cells can occur via Kupffer cell derived IL-12 and IL-18^[23,25]^, evidenced by the increased expression of activation markers CD69 and NKG2D and lower levels of inhibitory markers NKG2A^[26,27]^, but these are functionally suppressed^[28]^. These suggest that NK cells are unable to clear the infection on their own. The weakness of the innate response does not impair the induction of a vigorous HBV-specific CD4 T cell response^[29]^, that subsequently generates a large number of cytokines necessary for the effective development of cytotoxic CD8 T cells and B cell antibody production^[30]^. Potent HBV antigen-specific CD8^+^ T cell responses can control HBV replication and reduce it to undetectable levels during acute HBV infection^[31]^. In chronic HBV infection, the antiviral functionality of NK cells is also impaired, evidenced by an alteration of the phenotype and the receptors of NK cells^[32]^. This inhibition of NK cell activity is mainly mediated by myeloid-derived suppressor cells (MDSCs) via NKp30 receptor on NK cells^[33]^ and pro-inflammatory cytokines^[34]^. In addition, accumulated liver MDSCs due to HBV infection suppress CD8+ T cell function and promote systemic CD8+ T cell exhaustion^[35]^, characterized by high expression levels of inhibitory receptors such as CTLA-4, PD-1, and TIM-3^[36,37]^. Furthermore, they inhibit CD4+ T cells and metabolically regulate HBV-related liver damage^[38]^. MDSCs can induce the development of immunosuppressive regulatory T cells (Tregs) during chronic HBV infection primarily via a TGFb and the IL-10-dependent signalling pathway^[39]^. Tregs specifically inhibit CD8+ T cell activity; further blocking HBV-specific immune responses, leading to HBV persistence. On the other hand, low levels of HBV activity controlled by HBV antigen-specific CD8^+^ T cells lead to sustained liver inflammation and the functional depletion of HBV antigen-specific CD8^+^ T cells^[40–42]^. Hence, immunotherapies targeting these inhibitory receptors may modulate the progression of HCC [Figure 1]. Moreover, the exhausted CD8^+^ T cells experience impaired metabolic function and DNA repair capacity that further deteriorates their functions^[43]^. This highlights a complex interaction among the abovementioned immune cells during HBV infection, sustaining immune disorders and inflammation in the liver, which predispose patients to HCC development.

## HCV INFECTION AND IMMUNE TOLERANCE

The dysregulation in immune surveillance triggered by HCV infection is also thought to be one of the mechanisms by which HCV causes HCC. During acute HCV infections, NK cells are activated with enhanced cytotoxicity and IFN production^[44]^. However, 70% of HCV-infected patients progress to chronic infection^[45]^, partially due to decreased NK cell levels and function^[46]^. HCV antigen-specific CD8+ T cells participate in controlling HCV infection^[47]^. However, non-synonymous mutations in HCV are common, resulting in an escape from CD8+ T-cell recognition^[48,49]^. Moreover, HCV antigen-specific T cells undergo massive apoptosis during the chronic phase^[50]^. It has been reported that CD8+ T cell exhaustion develops following prolonged exposure to HCV antigens^[51–53]^. During chronic infection, HCV activates monocytes and macrophages, leading to the secretion of pro-inflammatory cytokines^[54]^. The released pro-inflammatory cytokines IL-6 and TNF not only promote macrophage apoptosis^[55]^, but also aggravate liver disease progression and HCC development^[56]^. In the setting of HCV infection, impaired macrophage phagocytosis may contribute to chronic infection and subsequent uncontrolled inflammation that promotes liver disease. Similar to HBV, HCV infection is also linked to the presence of MDSCs^[57]^ and an expansion of Tregs via IL-10^[58]^ and IL-12^[59,60]^. Tregs both suppress the HCV antigen-specific CD8+ T cell response in chronic infection and control memory cells. In addition, HCV impedes dendritic cell (DC) function by altering the adaptive response of CD4^+^ and CD8^+^ T cells, and cytokine release^[61,62]^. This suggests that HCV often disturbs antigen presentation along with humoral and cell-mediated immune response, resulting in chronic HCV infection and progressive liver damage [Figure 1].

## IMMUNE EVASION MECHANISMS OF HCC ASSOCIATED WITH HBV/HCV

Following persistent chronic liver inflammation due to HBV and HCV infection and immune imbalances, HCC develops with specific immunological features. There were 22% of 196 HCC samples displaying high or moderate levels of lymphocyte infiltration from an analysis of TCGA HCC samples, with high expression of immunosuppressive molecules and enriched Tregs, resting DCs and undifferentiated M0 macrophages compared to normal livers. This indicates an immunosuppressed microenvironment in this group of HCC patients. HBV/HCV infection status appeared not to be significantly associated with these observations^[63]^. There was also T-cell enrichment with heterogenetic clonal expansion of CD8^+^ T-cell populations with exhausted characteristics based on the sequencing of T-cell receptors (TCR) in TILs^[64,65]^. Interestingly, a further study showed CD8+ resident memory cells were enriched in HBV-related HCC with higher PD-1 expression and functionally more exhausted than non-virus-related HCC^[66]^. Increased numbers of CD14+ HLA-DR−/low MDSCs were found to be related to HCC progression^[67]^. Furthermore, infiltrating MDSCs not only suppress T-cell proliferation via arginase to deplete arginine^[67]^, but also promote Treg expansion through the production of IL-10 and TGF-β, and inhibit effector T cells through PD-L1^[67]^. In addition, high IL-10 secretion by MDSCs results in the skewing of resident tumour-associated macrophages (TAMs) and monocytes to an immunosuppressive phenotype^[68]^. They release TGF-β and VEGF to promote tumour growth and development, promoting cancer stem cells and metastasis^[69]^, stimulating Tregs, and suppressing NK cells^[70]^. Noticeably, Tregs are enriched in HCC^[64]^. This enrichment is prominent in HBV-related HCC with greater expression of PD-1 and increased suppressive function, which represents a more immunosuppressive and exhausted immune microenvironment in HBV-related HCC compared to the non-virus-related HCC^[66]^. The increased Tregs not only suppressed HBV antigen-specific immune responses, but also suppressed HCC tumour antigen-specific immune responses^[71]^. DCs are severely dysregulated in HCC, with a subset of CD14+ DCs expressing high levels of CTLA-4 which indicates an inhibitory phenotype^[72]^. In addition to these immune cells, several other stromal cells, such as NK cells, endothelial cells and cancer-associated fibroblasts, orchestrate immune evasion in HCC^[73]^. For example, endothelial cells in cancer tissues reportedly produce the C-X-C motif chemokine ligand 12, facilitating the recruitment of MDSCs^[74]^. Together, these data suggest that HCC is an immunogenic malignancy, rendering it an attractive target for immunotherapy [Figure 1].

## CURRENT IMMUNOTHERAPY OF HBV- AND HCV-RELATED HCC

Immunotherapy, specifically immune checkpoint inhibition, has been considered a useful treatment option for HCC, evidenced by both pembrolizumab (anti-PD-1) and nivolumab (anti-PD-1) with or without ipilimumab (anti-CTLA4) approved as second line therapy, and atezolizumab (anti-PD-L1) with bevacizumab approved as first line treatment options. In addition to immune checkpoint inhibitors (ICIs), several immunotherapy approaches are in development, including antibodies targeting specific tumour-associated antigens (TAAs), adoptive cell therapy, vaccination based on TAAs or mutation-associated neoantigens (MANAs) and oncolytic viruses. Although the infection of HBV and HCV is highly associated with HCC development, data on response outcomes specifically in this population included in trials is scarce.

## IMMUNE CHECKPOINT INHIBITORS

Tremelimumab, a CTLA-4 inhibitor, was the first immune checkpoint inhibitor (ICI) that showed encouraging results in patients with advanced HCC. In a phase II study including patients with advanced HCC and chronic HCV infection, tremelimumab showed an objective response rate (ORR) of 17.6%, a disease control rate (DCR) of 76.4%, a median time to progression of 6.48 months, and a median overall survival (OS) of 8.2 months^[75]^. Importantly, in this study, tremelimumab also exhibited antiviral effects evidenced by a significant decline in viral load. There were no treatment-related deaths and the treatment was mostly well tolerated.

In the open-label phase I/II CheckMate 040 trial, nivolumab was assessed as first-line therapy in patients with advanced HCC. The protocol had three concurrent cohorts of patients, including non-viral infected, HBV, and HCV infected advanced HCC. The results showed an ORR of 15%, a DCR of 58%, and a median OS of 15.6 months in the dose-escalation phase. The six-month OS was 83%, the nine-month OS was 74%, and the median duration of response (DOR) was 17 months in the dose-expansion phase. The most common treatment-related adverse events (TRAEs) were rash (23%) and pruritus (19%)^[76]^. Hepatitis flares were not reported. However, in the phase III randomized, double blind, multicentre CheckMate-459 trial, nivolumab failed to show statistical significance in OS benefit though there was a clear trend of improvement in OS for patients treated with nivolumab compared to sorafenib [Table 1]^[77]^. The viral infection history of the patient population remains unclear. Nivolumab is also being studied in the phase III CheckMate-9DX study as adjuvant treatment after curative therapy (surgery or ablation) for HCC in patients with a high risk of recurrence compared with placebo (NCT03383458). Recent reports from the combination of nivolumab and ipilimumab in patients with advanced or metastatic HCC showed an ORR of 33% with an 8% complete response (CR) among a total of 49 patients. There was a median DOR of 17 months with TRAEs of grade 3 or higher in 34% of patients. Among the trial cohort, 57% had an active HBV infection and 8% had an active HCV infection, and no evidence of viral hepatitis reactivation was detected^[68]^. Nivolumab and ipilimumab in the neoadjuvant setting (NCT03222076) have shown promising preliminary results of 29% pathologic CR with 34% TRAEs (5 HCV-positive and 1 HBV infected patients were reported).

Pembrolizumab is a recombinant monoclonal human antibody for human PD-1. A non-randomized, multicentre, open-label phase II study (KEYNOTE-224) tested the efficacy and safety of pembrolizumab in patients with advanced HCC as a second line treatment option, showing an ORR of 17% and a median OS of 12.9 months. HCV positive (*n* = 26) and HBV positive (*n* = 22) patients did not have reactivation of viral hepatitis^[78]^. However, the subsequent phase III randomized control trial KEYNOTE-240 of pembrolizumab as second line treatment in advanced HCC failed to show a statistically significant improvement in progression-free survival (PFS) or OS. Even so, pembrolizumab showed a reduced risk of death by 22% and an improved PFS compared with placebo. 25.9%, 15.5%, and 58.6% patients were affected by HBV, HCV, or non-infected in the pembrolizumab treatment cohort, respectively, in comparison to 21.5%, 21%, and 85% in the placebo cohort. A subgroup analysis indicated that patients with HBV infection treated with pembrolizumab had a superior median OS compared to those treated with placebo; there was no OS benefit in the group of HCV infected or non-infected patients^[79]^. There are two on-going phase III trials of pembrolizumab, including KEYNOTE-394, to evaluate pembrolizumab in Asian HCC patients, and KEYNOTE-937 to evaluate pembrolizumab as an adjuvant therapy in HCC patients after curative treatment.

Other PD-1 antibodies, including tislelizumab (BGB-A317), camrelizumab (SHR-1210) and cemiplimab (REGN2810), also have shown anti-tumour activity in HCC, with response rates of 16.7% (all responders were HBV infected)^[80]^, 13.8%^[81]^ and 19.2%^[82]^, respectively. Interestingly, in the trial of camrelizumab, 83% of patients enrolled were infected with HBV. An increase in HBV titre was noted in 46 participants, but the majority of these occurred after disease progression or after the last dose of treatment. Conversion to HBsAg positive from negative status was not reported during the treatment^[81]^. A phase III trial (RATIONALE 301) of tislelizumab versus sorafenib as first-line treatment in patients with unresectable HCC is currently underway (NCT02412773) [Table 2].

Durvalumab (MEDI4736) is an anti-PD-L1 monoclonal antibody. In a phase I/II trial of Child-Pugh class A advanced HCC patients, durvalumab achieved an OS rate of 10.3% in 39 patients in the second line setting. There was comparable ORR of 25% in patients with HCV infections and similar rates of TRAEs^[83]^. Furthermore, the combination of durvalumab with tremelimumab in patients with advanced HCC in the second line setting showed an ORR of 20% (2 responders were HCV infected), median PFS of 7.8 months, and median OS of 15.9 months amongst the 10 patients (7 HCV and 1 HBV infected)^[84]^. However, the other study reported this combination in advanced HCC showing no response in 9 patients with HCV infection, 1 responder in 11 patients with HBV infection, and an ORR of 35% among 20 uninfected patients with an overall ORR of 20%^[85]^. The on-going phase 3 HIMALAYA study evaluating durvalumab and tremelimumab compared with sorafenib or durvalumab monotherapy in the first-line setting in unresectable HCC (NCT03298451) may provide further information regarding the response status of HBV- or HCV-infected patients following anti-PD-L1 treatment.

It remains unknown whether virally-induced HCC is more prone to immune attack either secondary to the presence of foreign viral antigens or an immune response to the virus, compared to non-viral associated HCC. A recent pooled analysis assessed the efficacy of anti-PD1 or PDL1 in HBV infected HCC patients in comparison to non HBV infected HCC patients^[86]^. The results indicated that patients with HBV infection achieved ORRs similar to their non-infected counterparts, and this was seen with single and multi-agent treatment regimens. A lower disease control rate (DCR) was reported in HBV-infected HCC patients; stable disease was more likely to be seen in non-viral HCC, but this observation was not statistically significant. Drug efficacy evaluated as ORR and DCR of HCV-infected HCC patients compared to HBV positive HCC and non-viral HCC was similar, and reached statistical significance. Although clinical activity was observed for the most part in non-viral associated HCC patients, the interpretation of potential differences in response based on viral aetiology remains limited by the small number of patients and would require further evaluation with prospective, randomized, and double-blind clinical trials.

Given the profound immunomodulatory effect of the vascular epithelial growth factor (VEGF) pathway and dominant presence of angiogenesis in HCC, there increasing interest in testing the anti-tumour efficacy of ICIs in combination with anti-angiogenetic agents. For example, the anti-PD-L1 antibody atezolizumab was studied in a phase Ib study in combination with bevacizumab in the first-line setting for advanced HCC with Child-Pugh B liver disease^[87]^. This study showed promising early findings, resulting in an ORR of 34% with one CR^[87]^. This led to the multicentre, open-label, randomized phase III trial IMbrave 150, which evaluated this combination compared with sorafenib^[88]^. This study enrolled 336 patients; 49% were infected with HBV, 21% were infected with HCV, and 30% were non-viral in the combination cohort. In the sorafenib cohort 165 patients were enrolled; 46% had HBV, 22% had HCV, and 32% did not have hepatitis viral infections. The reported 12-month OS was 67.2% in the atezolizumab with bevacizumab group and 54.6% in the sorafenib cohort. Grade 3 or greater adverse events were reported in 56.5% of patients who received at least one dose of the combination treatment, and in 55.1% of patients in the sorafenib cohort. Interestingly, the subgroup analysis showed a superior OS benefit in patients with either HBV or HCV infection treated with combination therapy^[88]^. The FDA has approved the combination of atezolizumab and bevacizumab for the treatment of patients with unresectable HCC as a first line treatment option [Table 1]. There are other reports using ICIs in combination with anti-angiogenic therapies including pembrolizumab and lenvatinib^[89]^, durvalumab with ramucirumab^[90]^, nivolumab with ipilimumab and cabozantinib^[91]^, as well as avelumab with axitinib^[92]^. There are on-going trials with the same strategy, including atezolizumab and cabozantinib (COSMIC-312, NCT03755791), pembrolizumab with lenvatinib (LEAP-002, NCT03713593), SHR-1210 and apatinib (NCT03764293), and sintilimab (anti-PD-1) with bevacizumab biosimilar (ORIENT-32, NCT03794440).

The combined use of locoregional therapies such as ablation and transcatheter arterial chemoembolization (TACE) could improve the effectiveness of immunotherapies against HCC^[93]^. There are on-going phase III trials evaluating the outcome of the combination of ICIs with these modalities. For example, durvalumab and bevacizumab, or placebo with TACE in both intermediate HCC (EMERALD-1, NCT03778957) and high-risk HCC (EMERALD-2, NCT03847428), pembrolizumab with stereotactic body radiation therapy (NCT03316872), pembrolizumab following TACE (PETAL, NCT03397654) or Y90 (NCT03099564), and nivolumab with Y90 (NCT03033446).

Other immune checkpoint molecules, such as LAG3, TIM-3, 4–1BB, CD40, and OX40, can also be targeted and combined with PD-1/PD-L1 or CTLA-4 blockade in patients with HCC (NCT03005782, NCT03099109, NCT03241173). Biphasic antibodies to target PD-1 and other immune checkpoints concurrently are being studied as well (NCT03517488, NCT03752398).

## CELL-BASED IMMUNOTHERAPY

There are several cell-based immunotherapies being studied in patients with advanced HCC, including chimeric antigen receptor T (CAR-T) cells, cytokine-induced killer cells (CIKs) and T cell receptor (TCR)-engineered T cells.

Proteins found in HCC currently being investigated as targets in CAR-T cell research in early stage studies include GPC3 (NCT02905188, NCT03084380, NCT03130712, NCT03198546, and NCT03302403), AFP (NCT03349255), EpCAM (NCT03013712), c-Met/PD-L1 (NCT03672305), MUC-1 (NCT03198546), and DR5, c-Met or EGFRvIII (NCT03638206). The earliest study in CEA positive liver metastases treated with CAR-T cells was reported in a phase I trial. Anti-CEA CAR-T administered through hepatic artery infusion with or without systemic IL-2 treatment resulted in one case of stable disease (SD)^[94]^. HCC patients were not included, however. A phase I trial with anti-GPC3 CAR-T cells for relapsed or refractory GPC3-positive HCC showed one PR and three SD observed among 6 patients, respectively. No dose-limiting toxicity was identified and only one grade 3 fever was reported^[95]^.

Cytokine-induced killer cells (CIKs) are a mixture of heterogeneous immune cells generated by the *ex vivo* expansion of peripheral blood mononuclear cells with the support of IL-2, IFNg, and anti-CD3 monoclonal antibodies. A randomized phase II trial in treatment-naïve patients with HCC (over 50% patients had HBV infection) demonstrated that CIK therapy prolonged OS and PFS, compared to standard of care^[96]^. A multicentre open-label randomized phase III trial in patients with HCC after curative treatment demonstrated that CIK therapy prolonged recurrence-free survival and OS, though a significant proportion of patients with CIK infusion developed adverse events. In this trial, CIK infusion seemed to benefit the HBV-infected population (over 80% of the patient population) more than the HCV-infected or the uninfected group. No information of hepatitis flares or conversion was reported^[97]^.

TCR-engineered T cells are generated by integrating a cloned tumour antigen-specific TCR into T cells. Phase I trials are currently evaluating genetically modified T cells expressing AFP-specific TCRs in patients with advanced HCC (NCT03132792) and an autologous TCR-engineered T cell therapy targeting MAGEA1 in solid tumours including HCC (NCT03441100). Since HBV-DNA integration is often seen in HBV-related HCC, cell based therapy studies in HCC have looked into the possibility of using the HBV antigens expressed in HCC cells as a target for autologous TCR redirected therapy^[98,99]^. Vector-mediated gene transfer may be a means to introduce HLA-A2-restricted, HBV-specific TCRs into T cells of chronic HBV- and HBV-related HCC patients. Through TCR gene transfer, it has been demonstrated that TCR transduced T cells have the capacity of recognizing HCC cell lines expressing HBV antigens. This data showed that HBV-specific T cell clones cause apoptosis of HCC tumour cells that express the HBV X protein, proving that HBV proteins are identified by the immune system as non-self-tumour antigens^[100]^. Nevertheless, HBV antigens were expressed in HCC metastases and there is published evidence of the recognition of tumour cells by lymphocytes engineered to express HBV-specific receptor TCR with HCC autologous T cells genetically modified to express and HBV-specific TCR and treat chemo-resistant metastatic HCC^[101]^. These findings suggest that autologous TCR therapy redirected against HBV-associated HCC may have therapeutic potential in the future.

## VACCINES

Vaccines against HBV and HCV reduce the likelihood of developing HCC. Vaccine therapy in HCC is an area of important on-going research with the goal of improving the immune response against malignant cells through tumour specific antigens and subsequent T cell activation^[102]^. Clinical study protocols including different stages of HCC have been conducted by the Cancer Vaccine development for the HCC Consortium (HEPAVAC)^[103]^.

Both RNA and peptide-based vaccines are under investigation. A phase I/II trial for advanced solid tumours including HCC treated with NCI-4650, an mRNA-based vaccine, was terminated due to slow accrual (NCT03480152). Peptide-based vaccines for HCC utilize shared TAAs. A phase I trial evaluated the anti-tumour efficacy of an AFP-derived peptide vaccine subcutaneously injected in 15 patients with HCC; 10 HCV and 2 HBV infected patients. The study showed that the vaccine was well tolerated and 33% of the patients had an AFP-specific cytotoxic CD8+ T cell response. One patient had a CR for over 2 years and 8 patients had stable disease^[104]^. GPC3 is another antigen that is highly expressed in HCC. In a phase I trial of 33 patients (8 HBV and 15 HCV infected), the GPC3 peptide vaccine was well tolerated and induced a GPC3-specific T cell response. There was one PR (HCV infected) and 19 showing SD. GPC3-specific T cell frequency correlated with OS while higher GPC3-specific T cell frequency showed longer OS^[105]^. The additional PD-1 blockade seemed to augment the efficacy of the GPC3 vaccine by increasing the number of vaccine-induced cytotoxic T lymphocytes^[106]^. A phase II trial of a TERT-derived peptide vaccine in combination with low dose cyclophosphamide showed no effective antitumor response in 40 advanced HCC patients^[107]^. A study utilizing IMA970A with CV8102 vaccines has completed but the results have not yet been published (NCT03203005). Current vaccine trials include the hepcortespenlisimut-L vaccine (NCT02256514, NCT02232490), pneumonia vaccine (NCT03942328), heat shock protein-peptide complex vaccine (NCT04206254), Quilt-2.025 NANT neoepitope yeast vaccine (NCT03552718), DNAJB1-PRKACA fusion kinase peptide vaccine (NCT04248569), personalize DC vaccine (NCT03674073, NCT04147078) and multiple signals loaded DC vaccine (NCT04317248). The results of these trials will be instructive for the next generation of vaccine trial design.

## ONCOLYTIC VIRUSES

Oncolytic viruses have attracted lots of attention with the hope of tumour eradication through selective direct viral replication within tumour cells and activation of cell-mediated, tumour-specific immunity^[108]^. For example, JX-594 (Pexa-Vec, pexastimogene devacirepvec), derived from a strain of vaccinia, has been studied in HCC^[109,110]^. In a randomized phase 2 study with 20% HCV infected and 40% HBV infected patients among the 40 enrolled participants, JX-594 resulted in one CR and three PR^[109]^. Nevertheless, it also showed high-dose JX-594 doubled OS to 14.7 months from 6.7 months in the low-dose treatment group. All patients in the study experienced minimal TRAEs. In contrast, a phase 2b trial in 129 HCC patients in the second line setting, including 51.1% HBV- and 14.0% HCV-infected, did not show an OS benefit among 129 patients, compared to those treated with best supportive care^[111]^. Patients are presently being recruited for a clinical trial to test JX-594 with nivolumab (NCT03071094) and with sorafenib (NCT02562755), for treatment of advanced HCC as a first-line treatment.

## FUTURE DIRECTIONS

HCC is a heterogenic disease in terms of aetiology. HBV or HCV infection add to the complexity of the immune response in HCC. There are emerging data to illuminate the immune landscape, pathway, and mutation profiles of HCC that may provide aetiology-directed study design to obtain the best combination with immunotherapy in the future. Information about the specific immune and genetic landscape of HCV-related HCC is limited, however. In addition, the availability of reported response outcome from patients with different aetiologies in completed clinical trials would provide important data. The ultimate goal is to create aetiology-specific or even personalized therapies for HCC patients.

Furthermore, the schedule and sequence of this combination approach needs further evaluation to determine the optimal timing in order to obtain maximal tumour-directed immunological cell killing, whilst avoiding off-target effects. With more evidence available from other cancer types, especially haematological malignancies, utilizing a maintenance strategy versus moving to a first line or neoadjuvant approach for curative therapy in early HCC is also an interesting topic. In addition, along with the illumination of the effect of the gastrointestinal tract microbiome in HCC^[112]^, novel strategies in combination with antimicrobial therapy might be part of future treatment regimens (NCT03785210), such as chemotherapy, targeted therapy and radiation.

The overall clinical response to cell-based immunotherapy has not been robust, which indicates that this therapy may be more helpful when there is a lower disease burden or these precisely designed cells need to be used concurrently with other therapies in order to control HCC, e.g., in combination with ICIs. Moreover, there are subtle but substantial aspects of cell-based immunotherapy that need further evaluation, including virus antigen specific TCR therapy. A further example requiring better understanding is the mechanism by which trafficking of CAR-T cells into HCC cells to execute anti-tumour effects in situ can be achieved. This is a distinct problem observed in solid tumours that is not encountered in CAR-T technology in haematological malignancies.

Lastly, since the overall response to immunotherapy in HCC is suboptimal, it would be critical to identify responder candidates before treatment begins in order to improve the outcomes in patients with HCC associated with HBV or HCV infection. Though tumour mutation burden, PD-L1 expression, TILs, IFN signature and circulating tumour DNA have been indicated as predicative markers in other types of tumours, there has not been strong evidence showing that these markers are valuable in HCC. Therefore, further efforts to identify the predictive biomarkers that may help guide the selection of patients with HCC who are appropriate for ICIs are needed, such as microbiome and TCR repertoire targets. Along with this, intelligent, correlative studies from paired tumour biopsies will be helpful to identify the best therapeutic approaches, timing, and sequences, and improve outcomes of patients with HCC.

## Figures and Tables

**Table 1.** Approved treatments for advanced HCC

Treatment	Benefit	Level of evidence	Comments
Atezolizumab and bevacizumab^[78]^	↑ survival	1A	Non-curative treatment, superior to first line sorafenib
			In unresectable HCC, bevacizumab-atezolizumab has a better OS and PFS compared to sorafenib
			Improved OS with bevacizumab-atezolizumab at 6 months (84.8%) and 12 months (67.2%) *vs.* 72.2% and 54.6% respectively with sorafenib
			PFS is longer with atezolizumab-bevacizumab (median 6.8 months) than with sorafenib (median 4.3 months)
			Most common grade 3 or 4 AEs: hypertension, AST increase, ALT increase, fatigue, proteinuria, diarrhoea, decreased appetite, pyrexia
Nivolumab^[67]^	No survival benefit	1A	Treatment of advanced HCC previously treated with sorafenib
			Durable ORR of 14%, median duration of response 17 months
			Median OS as second-line therapy:15.6 months, non-curative
			Well-tolerated
			In front line setting *vs.* sorafenib did not show increase in OS (phase III study)
Pembrolizumab^[70]^	No survival benefit	1A	Treatment of advanced HCC previously treated with sorafenib
			Overall durable response rate of 17%, PFS 4.9 months, non-curative treatment
			Well tolerated
Nivolumab and ipilimumab^[68]^	↑ survival	1A	Treatment of advanced HCC after failure of sorafenib treatment
			Objective response 31%, median duration of response 17 months
			Most common AEs: fatigue, diarrhoea, rash, pruritus, nausea, musculoskeletal pain, pyrexia, cough, decreased appetite, vomiting, abdominal pain, dyspnoea, upper respiratory tract infection, arthralgia, headache, hypothyroidism, decreased weight, dizziness
			More than 50% of patients may require systemic steroids to manage AEs

Evidence-based classification adapted from the National Cancer Institute. 1 = Randomized controlled trial or meta-analysis; 2 = Non-randomized controlled trial; 3 = Case series; A = Survival endpoint; B = Cause-specific mortality; C = Quality of life; D = Indirect surrogates. OS: overall survival; AEs: adverse events; HCC: hepatocellular carcinoma; ORR: objective response rate; AST: aspartate transaminase; ALT: alanine aminotransferase

**Table 2.** Key immunotherapy trials

Drug name	Trial number	Phase	Comments
Camrelizumab^[72]^	NCT02989922	II	Response rate 14%. Median OS 14.4 months (predominantly HBV-positive patients)
Tislelizumab	NCT02412773	III	Active recruiting
Pembrolizumab (Keynote 937)	NCT03062358	III	Active recruiting in Asia
Pembrolizumab (Keynote 394)	NCT03062358	III	Active accrual in Asia
Nivolumab (Checkmate 9DX)	NCT03383458	III	Currently recruiting
Nivolumab and Ipilimumab	NCT03222076	II	Currently recruiting
Cemiplimab^[73]^	NCT03916627	II	Currently recruiting
Tislelizumib	NCT03412773	III	Results pending
Durvalumab with tremelimumab and ablation^[75]^	NCT02821754	I/II	Response rate 20% Median PFS 7.8 months
Durvalumab with tremelimumab (HIMALAYA )	NCT03298451	III	Currently recruiting

PFS: progression-free survival; OS: overall survival
